# High glucose-induced p66Shc mitochondrial translocation regulates autophagy initiation and autophagosome formation in syncytiotrophoblast and extravillous trophoblast

**DOI:** 10.1186/s12964-024-01621-x

**Published:** 2024-04-20

**Authors:** Lulu Ji, Xiaoli Zhang, Zhiguo Chen, Yuexiao Wang, Hengxuan Zhu, Yaru Nai, Yanyi Huang, Rujie Lai, Yu Zhong, Xiting Yang, Qiongtao Wang, Hanyang Hu, Lin Wang

**Affiliations:** 1 Department of Histology and Embryology, TaiKang Medical School (School of Basic Medical Sciences), Wuhan University, Hubei Province, Wuhan, 430071 China; 2 Department of Ultrasound in Gynecology and Obstetrics, Zhongnan Hospital of Wuhan University, Hubei Province, Wuhan, 430071 China; 3 Department of Human Anatomy, Basic Medical Sciences of Xinxiang Medical University, Henan Province, Xinxiang, 453003 China; 4 Hubei Provincial Key Laboratory of Developmentally Originated Disease, Hubei Province, Wuhan, 430071 China

**Keywords:** High glucose, p66Shc, Autophagy, cGAS/STING, MAM

## Abstract

**Background:**

p66Shc, as a redox enzyme, regulates reactive oxygen species (ROS) production in mitochondria and autophagy. However, the mechanisms by which p66Shc affects autophagosome formation are not fully understood.

**Methods:**

p66Shc expression and its location in the trophoblast cells were detected in vivo and in vitro. Small hairpin RNAs or CRISPR/Cas9, RNA sequencing, and confocal laser scanning microscope were used to clarify p66Shc’s role in regulating autophagic flux and STING activation. In addition, p66Shc affects mitochondrial-associated endoplasmic reticulum membranes (MAMs) formation were observed by transmission electron microscopy (TEM). Mitochondrial function was evaluated by detected cytoplastic mitochondrial DNA (mtDNA) and mitochondrial membrane potential (MMP).

**Results:**

High glucose induces the expression and mitochondrial translocation of p66Shc, which promotes MAMs formation and stimulates PINK1-PRKN-mediated mitophagy. Moreover, mitochondrial localized p66Shc reduces MMP and triggers cytosolic mtDNA release, thus activates cGAS/STING signaling and ultimately leads to enhanced autophagy and cellular senescence. Specially, we found p66Shc is required for the interaction between STING and LC3II, as well as between STING and ATG5, thereby regulates cGAS/STING-mediated autophagy. We also identified hundreds of genes associated several biological processes including aging are co-regulated by p66Shc and ATG5, deletion either of which results in diminished cellular senescence.

**Conclusion:**

p66Shc is not only implicated in the initiation of autophagy by promoting MAMs formation, but also helps stabilizing active autophagic flux by activating cGAS/STING pathway in trophoblast.

**Supplementary Information:**

The online version contains supplementary material available at 10.1186/s12964-024-01621-x.

## Background

Gestational diabetes mellitus (GDM) is a prevalent chronic condition during pregnancy that impairs the health of millions of women worldwide and increases the risk for adverse maternal and neonatal outcomes. The etiology of GDM is varied, and dysregulation of placental hormones has been traditionally connected to insulin sensitivity [1]. But the cause of placental dysfunction is still unclear in GDM patients. Autophagy is a conserved process in eukaryotes that maintains cellular homeostasis during environmental stress. It plays a crucial role in trophoblast functions, such as invasion and vascular remodeling in extravillous trophoblasts (EVTs), for normal placental development [2]. We previously reported damaged mitochondria and increased autophagy in trophoblast cells of GDM placentae [3], but the underlying molecular mechanism remain elusive.

p66Shc is a member of ShcA protein family. Several stimuli can induce expression and serine phosphorylation of p66Shc, enabling its translocation to mitochondrial intermembrane space. The process subsequently leads to the generation of mitochondrial reactive oxygen species (ROS) and initiates mitochondrial dysfunction [4]. Although the formation of a complex between p66Shc and LC3-II, and high expression of p66Shc in placentae of GDM are well-documented [5, 6], the role of p66Shc in initiating autophagosome formation remains elusive. The sites of interaction between the endoplasmic reticulum (ER) and mitochondria (Mito), known as MAMs, serve as crucial initiation sites for autophagy, a fundamental molecular pathway essential for maintaining cellular and organismal homeostasis [7]. Aberrant formation of MAMs represents a pivotal step leading to mitochondrial dysfunction and have been implicated in disease, including Alzheimer disease, cancer, diabetes mellitus, obesity-induced mitochondrial dysfunction, and metabolic disorders [8]. The tethering of MAMs is facilitated by several mitochondrial molecular bridge, such as VDAC1, Mfn2 and FUN14 et al, [8–10]. However, whether mitochondrial translocation of p66Shc regulates MAM formation in trophoblast cells remains elusive.

Recently, cGAS/STING pathway serves as a key step in autophagosome biogenesis [11, 12]. cGAS (cyclic GMP-AMP synthase) is a cytosolic DNA sensor, which activates STING (stimulator of interferon genes) through the second messenger molecule cGAMP (2′3’-cGMP-AMP) [13]. High glucose induces mitochondrial dysfunction, mtDNA fragmentation, leading to the release of damaged mtDNA into the cytosol in keratinocytes [14], potentially triggering activation of the cGAS/STING pathway. Although we previously found changes in mitochondrial ultrastructure and mitophagy, it remains unclear if p66Shc is associated with mtDNA release into the cytosol to promote cGAS/STING-mediated autophagosome formation in trophoblast cells.

Here, we examined the role and distribution of p66Shc in syncytiotrophoblast (STBs) and extra villous trophoblast (EVTs) cells in vivo and in vitro. Furthermore, we investigated the location of p66Shc and its effects on autophagy initiation and autophagosome formation. We found exposure to high glucose promoted the formation of MAMs, enhanced p66Shc’s localization in mitochondria, facilitated mtDNA release into cytoplasm, and the activated cGAS/STING pathway. Moreover, depletion of p66Shc significantly ameliorate cellular dysfunctions described above. Besides, we demonstrated that knockdown of p66Shc attenuated the interaction between STING and LC3-II, as well as between STING and ATG5. Our findings provide novel insight into p66Shc regulated autophagy and cellular function of STB and EVT cells in high glucose stress.

## Methods

### Patients

Twenty patients diagnosed with GDM and 27 healthy pregnancy women were recruited from the Department of Gynecology and Obstetrics in The Central Hospital of Wuhan between 2016 and 2018. The maternal serum Mn-SOD were obtained from the medical record room in the Zhongnan Hospital of Wuhan University during 2012 to 2017. The participants in the study all experienced uncomplicated singleton pregnancies. The GDM patients did not receive any prenatal pharmacological intervention and exhibited inadequate glycemic management throughout pregnancy. A diagnosis of GDM was based on the 75g oral glucose tolerance test (OGTT), together with the IADPSG (International Association of Diabetes and Pregnancy Study Group) criteria. Blood samples were collected when screening for GDM using OGTT at 24–28 weeks of gestation. The study was approved by the Ethics Committees of the Wuhan University School of Medical Sciences. All participants were fully informed about the study and provided written informed consent.

### Cell culture

The human trophoblast cell line HTR8/SVneo was purchased from American Type Culture Collection and cultured in RPMI 1640 medium supplemented with 10% fetal bovine serum (FBS, Gibco, USA), 100 U/mL penicillin and 100ng/mL streptomycin (Gibco, 15140) at 37 ℃ in a 5% CO_2_ incubator. The cells were treated with 5 mM or 30 mM glucose and then cultured in a high-glucose environment for 24 hours and collected for further analysis.

Bewo cells are a human trophoblast-derived choriocarcinoma cell line, which used to model syncytialization of villous trophoblasts. Bewo cells were cultured in F-12 K medium containing 10% FBS and 1% penicillin and streptomycin 37 ℃ in a 5% CO_2_ incubator. The cells were treated with 5 mM or 30 mM glucose and then cultured in a high-glucose environment for 24 hours and collected for further analysis.

### Plasmid construction and transient transfection

The human p66Shc cDNA was amplified by PCR and subsequently inserted into the HindIII and XbaI restriction sites of the pcDNA3.1(+) expression plasmid (p66WT), or alternatively cloned into the NheI and EcoRI sites of the pcDNA3.1-EGFP expression plasmid (GFP-p66Shc). Q5 Site-Directed Mutagenesis Kit (NEB) was utilized for the generate of p66Shc mutants (p66SC and p66QQ). The pBABE-puro-mCherry-EGFP-LC3B plasmid was purchased from Addgene. pDsRed2-Mito was purchased from MiaoLing Plasmid Platform. Then the vectors with target gene were respectively transfected into HTR8/SVneo cells using the Lipofectamine 2000 reagent (Invitrogen) according to the manufacturer’s instructions. The recombinant DNA used in this study are listed in Supplementary Table S1.

### Knockdown and knockout of target genes in HTR8/SVneo cells

The lentiviral shRNA constructs targeting the human p66Shc and control were cloned into pLKO.1 plasmid. shRNA containing pLKO.1 vector was co-transfected with psPAX2 and pMD2.G into 293T cells using PEI transfection reagent (Polysciences, 26406). The viruses were collected at 48- and 72-hours post-transfection, followed by their utilization for cell infection in the presence of Polybrene (8 µg/mL, Sigma). Stable transfectants were generated by limiting dilution after selection with Puromycin (MCE) for 2 weeks. The shRNA sequences of p66Shc (p66Shc-KD) and control are provided in Supplementary Table S2.

The lentiviral sgRNA constructs targeting the human STING (sgSTING1/2) and control (sgNC) were cloned into the LentiCRISPRv2 plasmid, and co-transfected with psPAX2 and pMD2.G into 293T cells using PEI. Viruses were collected at 48-and 72-hours post-transfection, followed by their utilization for cell infection in the presence of Polybrene (8 µg/mL, Sigma). Stable transfectants were generated by limiting dilution after selection with Puromycin (MCE) for 2 weeks. The sgSTING1/2 and sgNC sequences are provided in Supplementary Table S2.

Small interfering RNA against ATG5 and negative control siRNA were transfected using the jetPRIME transfection reagent (Polyplus Transfection). The sequences for siRNAs are provided in Supplementary Table S2.

## RNA isolation, qRT–PCR, Western blot, Co-Immunoprecipitation

### Mitochondrial protein extraction, MMP measurement and TEM

Total RNA extraction, complementary DNA synthesis, and qRT-PCR were performed as previously described [3]. Primer sequences of qRT-PCR were provided in Supplementary Table S1. Placental tissues and cell lines were processed for western blot as previously described [3]. The band intensity on the western blots was quantified using Image J software. Control and p66Shc-KD cells were performed to co-immunoprecipitation as previously decreased [15]. Antibodies were shown in Supplementary Table S3. The Tissue Mitochondria Isolation Kit (Beyotime, C3606) and Cell Mitochondria Isolation Kit (Beyotime, C3601) were utilized for the extraction of mitochondrial proteins from placentae and trophoblast cell line, according to the manufacturer’s protocol. HTR8/SVneo, control and p66Shc-KD cells were treated with 30 mM glucose for 24 hours. Subsequently, the Mitochondrial membrane potential assay kit with JC-1 (Beyotime, C2006) was employed to measure MMP levels in accordance with the manufacturer’s instructions. Then the fluorescent intensity of both JC-1 monomers and aggregates was monitored by flow cytometry.

For TEM, cells were harvested and subjected to centrifugation at 1500 rpm for 5 minutes. Subsequently, the cellular pellets and placental tissues (1 mm^3^/piece) were fixed in a solution containing 2.5% (v/v) glutaraldehyde in 0.1 M phosphate buffer (pH 7.4) for a duration of 2 hours at room temperature (RT). Then the collected samples were sent to our Research Center for Medical and Structural Biology for further processing. Observations of 10 independent cells per condition were carried out on a Hitachi HT7700 electron microscope at 100 kV.

### Immunohistochemistry

Placental tissues from NP and GDM patients were embedded in paraffin and sliced into 5 μm sections. The sections were subsequently dewaxed, rehydrated, and subjected to antigen retrieval by incubation in 10 mM citrate buffer at 95 °C. After blocking with a 5% BSA solution at 37°C for one hour, the placental tissue sections were incubated overnight with the indicated primary antibodies at 4°C. The sections underwent dewaxing, rehydration, and antigen retrieval by incubation in 10 mM citrate buffer at 95 °C. Following blocking with a solution of 5% BSA at 37 °C for an hour, the placental tissue sections were incubated overnight with indicated primary antibodies at 4 °C. After being washed three times with PBS, the sections were incubated with secondary antibody at 37°C for one hour. Finally, diaminobenzidine was used for sample staining and Mayer haematoxylin was employed for counterstaining before capturing images from five fields of each section.

### RNA-seq and data analysis

Total RNA from p66Shc-KD, ATG5-KD, DKD and control cells were extracted using Trizol. RNA-seq libraries were prepared for paired-end Illumina NovaSeq 6000 using a 2x150-bp paired-end configuration. All raw RNA-seq data were mapped to hg19 using Subread package with reference gene annotation GENCODE, release 19. The alignment BAM files were subjected to read counting using the featureCounts package. edgeR package was used for all differential expression analysis from the raw counts. The read counts were converted into RPKM (reads per kilobase of exon model per million mapped reads) using edgeR package. Gene set enrichment analysis was performed using GSEA software. Pathway analysis were performed using DAVID database. Expressions of genes were visualized as heatmaps in a multiple experiment viewer (MeV, version 4.9.0).

### Public microarray data

Publicly available microarray data for NP and GDM placentae were obtained from GSE128381. Differentially expressed genes (DEGs) were determined using the limma R package. Pathway analysis were performed using DAVID database.

### Measurement of mitochondrial DNA release

The cells were seeded into a 6-well plate. After the designated treatment, 1% NP40 was added to each well and the cells were scraped. The wells were supplemented with 1% NP40 and the cells were harvested by scraping. Place lysates into prelabeled microcentrifuge tubes and incubate on ice for 15 min, spin lysates at 13,000 rpm (16,000 x g) for 15 min at 4 °C to pellet the insoluble fraction as described [16]. The supernatant (the cytosolic fraction) was utilized for the isolation of cytosolic mitochondrial DNA using the TIANamp Genomic DNA Kit (TIANGEN, DP304). The purified DNA were then subjected to qPCR analysis. Primers used for mitochondrial genes (mt gene) and internal control (18S rDNA, internal control) are listed in Supplementary Table S1.

### Mitochondrial ROS measurement and SA-β-gal activity assay

The measurement of mitochondrial ROS was conducted using MitoSOX as per the manufacturer's instructions. Briefly, cells were incubated with MitoSOX reagent (5 μM; Thermo Fisher Scientific, M36008) for 10 minutes at 37 °C. After incubation, the cells were washed three times with PBS and fluorescence microscopy was employed to measurement the superoxide generated in the mitochondria of live cells.

The SA-β-gal activity assay was performed using the Senescence β-Galactosidase Staining Kit (Beyotime #C0602) according to the manufacturer’s instructions. In brief, cells cultured in 6-well plates were thoroughly washed, fixed, and subsequently stained with X-gal solution overnight at a temperature of 37 °C. Subsequently, the cells were visualized and captured using a microscope.

### Statistical analyses

Unpaired two-sided Student's t-tests were utilized for appropriate comparisons between two groups. The error bars indicate the standard error of the mean. The individuals conducting laboratory experiments were unaware of the clinical data pertaining to the patients, and blinding procedures were implemented in animal studies. All experiments were repeated a minimum of three times for each test, ensuring technical accuracy. GraphPad Prism 8 software (San Diego, USA) was used for all statistical analyses and p < 0.05 was considered statistically significant.

## Results

### High glucose reinforced the expression and mitochondrial translocation of p66Shc

**Fig. 1:**
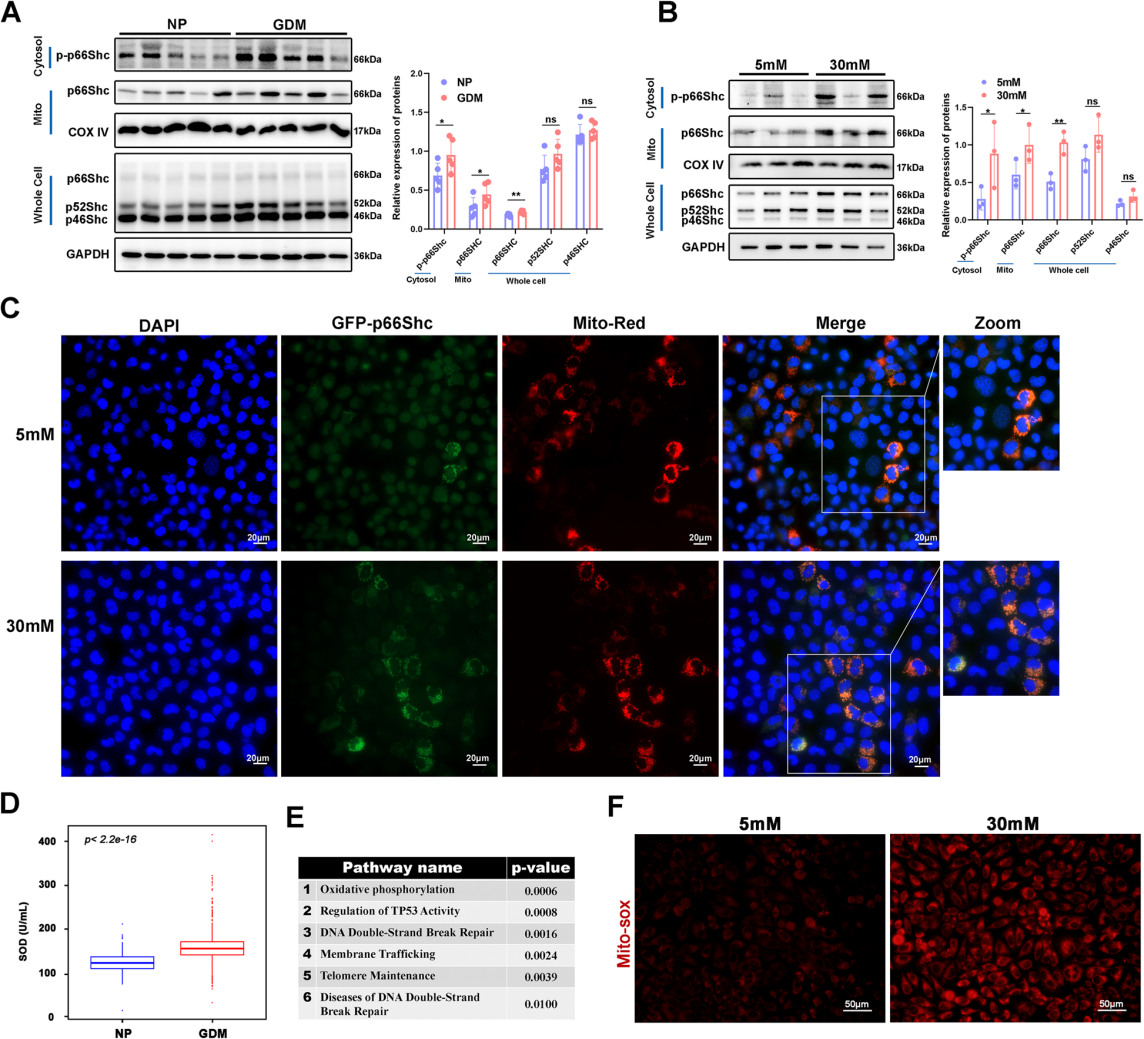
High glucose increased expression of p66Shc and its translocation to mitochondrion. **A** Western blot images and quantifications of protein expressions of p-p66Shc in cytosol, p66Shc in mitochondria and whole cell lysate in NP and GDM placentae. *P < 0.05, **P < 0.01. **B** Western blot images and quantifications of protein expressions of p-p66Shc in cytosol, p66Shc in mitochondria and whole cell lysate in 5 mM or 30 mM glucose treated HTR8/SVneo cells. *P < 0.05, **P < 0.01. **C** Fluorescence microscope images of co-localization of p66Shc (green) with mitochondria (red) in 5 mM or 30 mM glucose treated HTR8/SVneo cells. Scale bar, 20 μm. **D** Box plot shows the levels of maternal serum Mn-SOD between NP (*n* = 281) and GDM patients (*n* = 1037). **E** Gene ontology analysis of differentially expressed genes between NP and GDM placentae. **F** MitoSOX staining showing levels of mitochondrial superoxide in 5 mM or 30 mM glucose treated HTR8/SVneo cells. Scale bar, 50 μm

We first examined the expression and subcellular location of p66Shc to determine its role in GDM. We obtained highly pure mitochondria from placentae, and observed strong expression of p66Shc in mitochondrial lysate and p-p66Shc in cytoplasmic lysate of GDM patients (Fig. 1A). Similarly, high glucose also increased the expression of p66Shc and p-p66Shc in mitochondrion and cytosol of HTR8/SVneo cells, respectively (Fig. 1B). We also detected increased expression and mitochondrial translocation of p66Shc in Bewo cells (Figure S1A). Besides, significantly higher levels of p66Shc mRNA were also observed in trophoblast cells under high glucose both in vivo and in vitro (Figure S1B-C). We further observed enhanced mitochondrion translocation of p66Shc in HTR8/SVneo and Bewo cells under high glucose condition, indicated by the colocalization of GFP tagged p66Shc and mitochondria-originating fluorescent signals (Fig. 1C and Figure S1D). These observations suggest high glucose increased expression and mitochondrial translocation of p66Shc in trophoblast cells, and p66Shc may contribute to oxidase damage of GDM placentae.

Indeed, we found level of manganese-dependent superoxide dismutase (Mn-SOD) in maternal serum is significantly increased by 30% in GDM group with a higher BMI (Fig. 1D and Figure S2A), suggesting more severe oxidative stress injury in GDM patients. Additionally, Mn-SOD level is positively correlated with BMI but not age (Figure S2B-C), indicating that peripheral blood SOD levels in pregnant women may serve as a partial indicator of maternal oxidative stress associated with GDM. We then analyzed public gene expression microarray from NP and GDM placentae (GSE128381) of term birth to identify affected molecular pathways. We observed dysregulated gene signatures for several pathways including the oxidative phosphorylation (*p* = 0.0006), regulation of TP53 activity (*p* = 0.0008), DNA double strand break repair (*p* = 0.0016) and Telomere maintenance (*p* = 0.0039) (Fig. 1E). Oxidative phosphorylation occurs in the mitochondria, and is a process for the production of ROS. Consistently, we observed that mitochondrial superoxide was promoted by high glucose (Fig. 1F). These findings demonstrated strong connection between p66Shc and placental oxidative stress and dysfunction.

### p66Shc facilitates autophagy in trophoblast cells

**Fig. 2:**
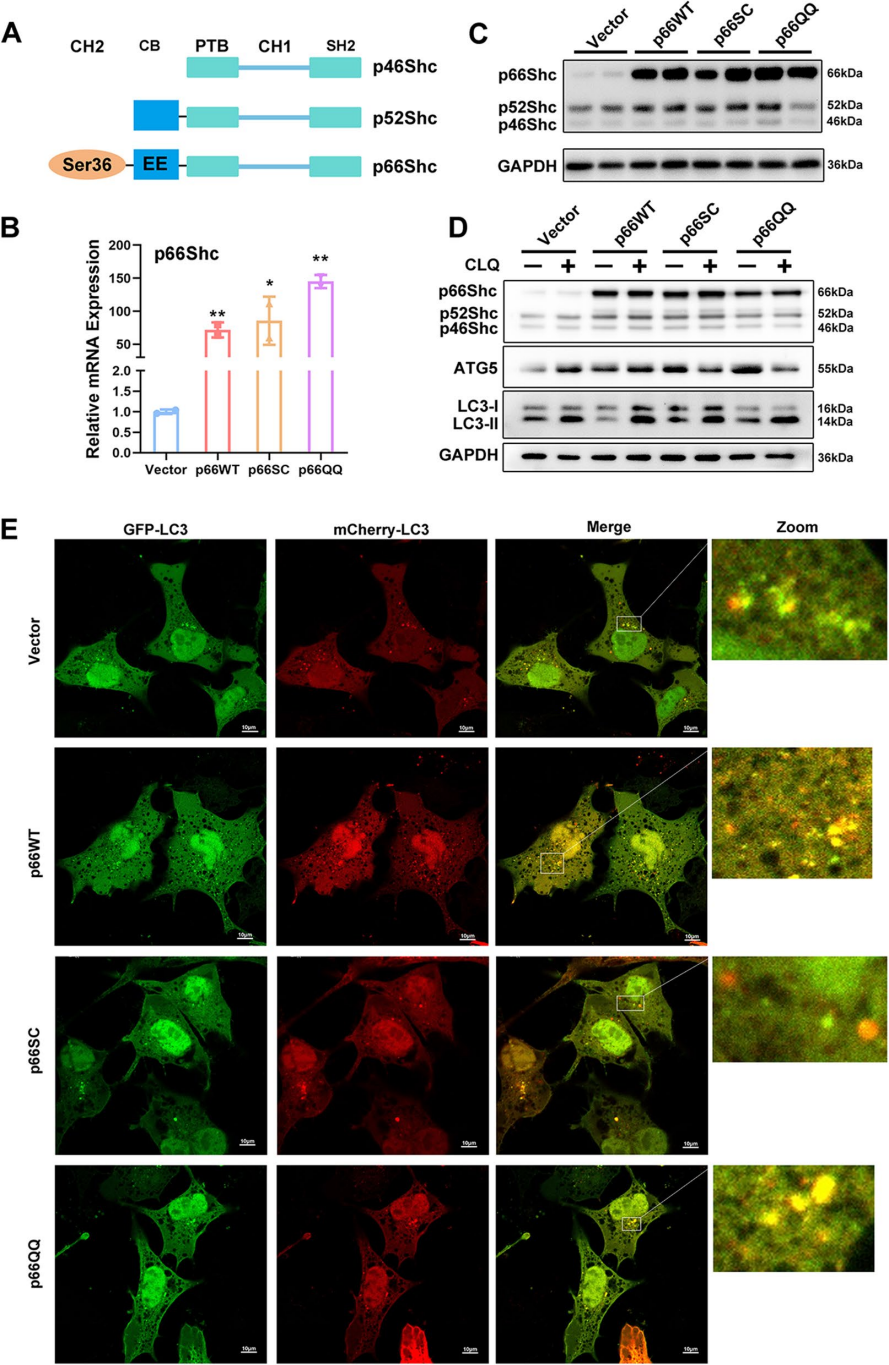
p66Shc promotes autophagy in trophoblast cells. **A** Schematic structure representations of three isoforms for p46Shc, p52Shc and p66Shc, and the locations of phosphorylatable serine at position 36 in the CH2 domain and two glutamic acid residues at positions 132 and 133 in the CB domain. **B**-**C** qRT-PCR analysis of *p66Shc* mRNA expression **B** and Western blot images of p66Shc protein expression **C** in p66WT, p66SC and p66QQ cells. *P < 0.05, **P < 0.01. **D** Western blot images of protein levels of p66Shc, ATG5 and LC3 in p66WT, p66SC and p66QQ overexpressed HTR8/SVneo cells treated with Chloroquine. **E** Representative confocal images of autophagic flux were obtained from HTR8/SVneo cells overexpressing p66WT, p66SC, and p66QQ and transfected with mCherry-EGFP-LC3 to label the autophagosomes (yellow) and autolysosomes (cherry). Scale bar, 10 μm

ROS generation by p66Shc relies on two different domains of the protein [5]. One is CH2 domain containing a phosphorylatable serine at position 36 (S36), the other is CB domain with two glutamic acid residues at positions 132 and 133 (Fig. 2A). Therefore, we constructed two p66Shc mutants called p66SC (carrying a C substitute for S at position 36) or p66QQ (carrying QQ substitute for EE at positions 132 and 133) (Figure S3A). We overexpressed wild-type p66SHC (p66WT), p66SC and p66QQ to probe the effects of p66Shc on autophagy in trophoblast cells. As is showed in Fig. 2B-C, p66Shc were significantly overexpressed on both mRNA and protein levels, indicating the successful overexpression of three types of p66Shc in HTR8/SVneo cells.

We next examined autophagic flux in the three types of cells. Western blotting results revealed that the enhancement in autophagic flux in the presence of p66WT was abrogated in cells expressing p66SC, but not p66QQ, despite a decrease in ATG5 levels observed in both p66SC and p66QQ cells (Fig. 2D). Furthermore, we observed an increase in the number of autophagosomes in the presence of p66WT and p66QQ compared with p66SC, as determined by imaging cells transiently transfected with mCherry-EGFP-LC3 vector (Fig. 2E). The present data suggest that the upregulation of autophagy induced by high glucose is attributed to the elevation and mitochondrial translocation of p66shc.

### p66Shc knock-down rescues high glucose induced autophagy and senescence of trophoblast cells

**Fig. 3:**
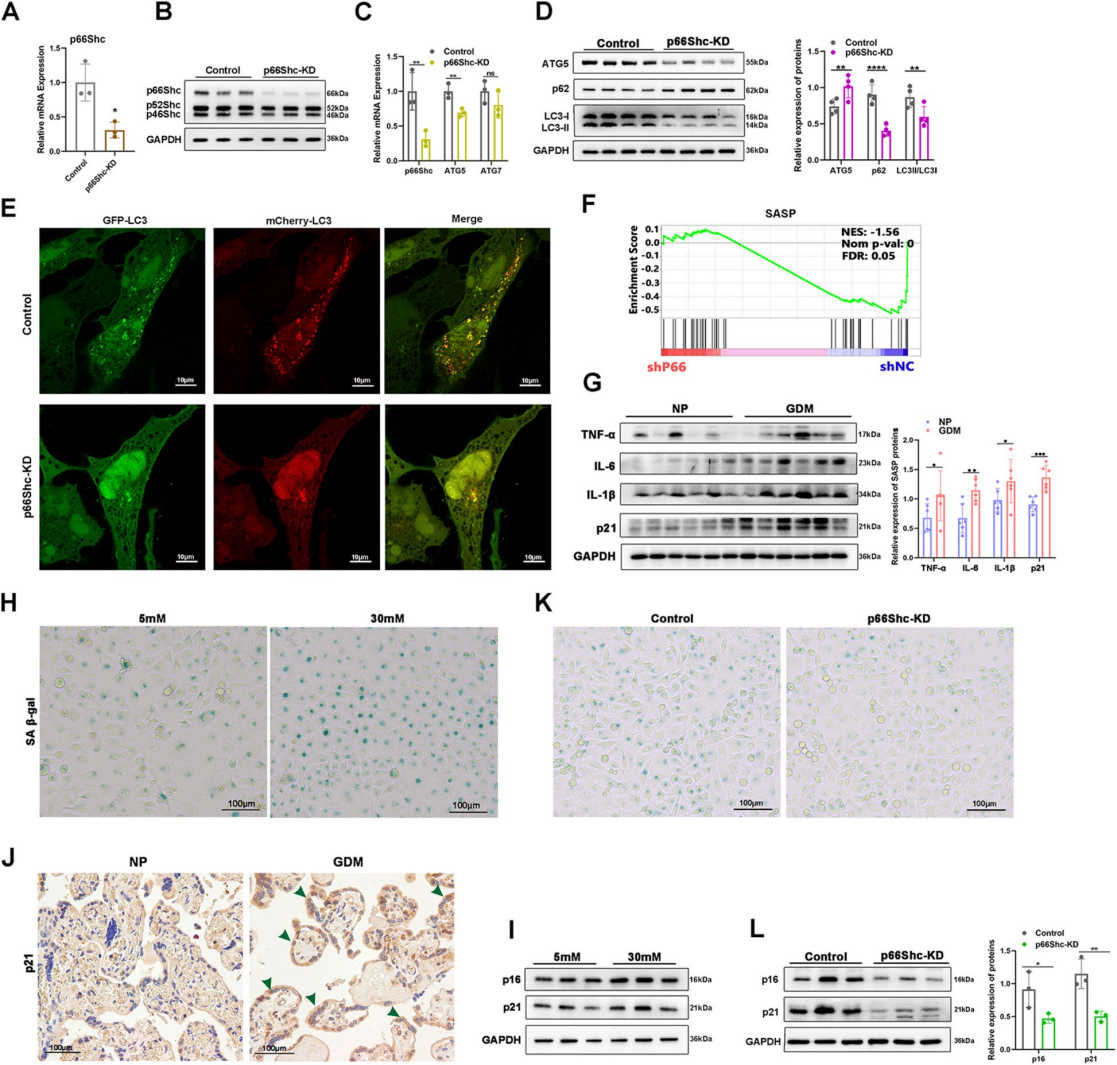
p66Shc knock-down rescues high glucose induced autophagy and senescence of trophoblast cells. **A**-**B** qRT-PCR analysis of *p66Shc* mRNA expression **A** and Western blot images of p66Shc protein expression (B) in control and p66Shc-KD cells. **C** qRT-PCR analysis of *p66Shc*, *ATG5* and *ATG7* mRNA expressions in control and p66Shc-KD cells. **D** Western blot images and quantifications of protein levels of ATG5, p62 and LC3 in control and p66Shc-KD cells. **E** Representative confocal images of autophagic flux in control and p66Shc-KD cells transfected with mCherry-EGFP-LC3 to label the autophagosomes (yellow) and autolysosomes (cherry). Scale bar, 10 μm. **F** Gene set enrichment analysis of SASP gene signature in control and p66Shc-KD cells. **G** Western blot images and quantifications of SASP protein expressions of TNF-α, IL-6, IL-1β and p21 in placentae of NP and GDM patients. *P < 0.05, **P < 0.01, ***P < 0.001. **H** SA-β-gal staining images showing cell senescence in 5 mM or 30 mM glucose treated HTR8/SVneo cells. Scale bar, 100 μm. **I** Western blot images of p16 and p21 protein levels in 5 mM or 30 mM glucose treated HTR8/SVneo cells. **J** IHC staining images of p21 in NP and GDM placentae. Green arrowheads indicate upregulation of p21 both in the cytoplasm and nucleus, with a predominant expression in the cytoplasm of syncytiotrophoblast cells in GDM placentae. Scale bar, 100 μm. **K** SA-β-gal staining showing cell senescence of control and p66Shc-KD cells. **L** Western blot images and quantifications of protein levels of p16 and p21 in control and p66Shc-KD cells. *P < 0.05, **P < 0.01, ****P < 0.0001

To further explore whether p66Shc is critical to autophagy in trophoblast cells of high glucose, we constructed p66Shc stable knockdown HTR8/SVneo cell line (p66Shc-KD) (Fig. 3A-B). Repression of p66Shc resulted in a reduction in the mRNA levels of *ATG5*, while no significant change was observed in the mRNA levels of *ATG7* (Fig. 3C). The protein levels of ATG5 and the ratio of LC3II/LC3I were found to be significantly reduced, concomitant with an increased expression of p62 (Fig. 3D). Moreover, the number of autophagosomes and autolysosomes were also decreased in p66Shc-KD cells (Fig. 3E). Hence, p66Shc enhances high glucose induced autophagy in trophoblast cells.

We determined the global gene expression alterations after perturbation of p66Shc in high glucose-treated HTR8/SVneo cells by RNA-seq. Gene set enrichment analysis (GSEA) revealed that the inhibition of p66Shc in the suppression of genes associated with senescence associated secretory phenotype (SASP), a hallmark of senescence (Fig. 3F), indicating a critical role of p66Shc in senescence regulation. We measured the level of SASP proteins in GDM placentae and found increased levels of TNF-α, IL-6, IL-1β and p21 (Fig. 3G), with unchanged p16, MCP1, MMP3 and PAI (Figure S3B). Cell senescence was also measured by senescence-associated β-galactosidase (SA β-gal) activity, another hallmark of senescence. Similarly, high glucose treatment increased the SA β-gal levels and also elevated protein levels of p16, p21 in HTR8/SVneo cells (Fig. 3H-I). In addition, Immunohistochemistry (IHC) analysis indicated that p21 level was also markedly increased in the syncytiotrophoblast of GDM patients (Fig. 3J), and Western blot also showed increased p16 and p21 in Bewo cells (Figure S3C). In contrast, inhibition of p66Shc diminished the SA β-gal activity and protein levels of p16 and p21 (Fig. 3K-L). These findings indicated that elevated levels of p66Shc in trophoblast cells exposed to high glucose led to enhanced autophagy and induction of cellular senescence.

### p66Shc and ATG5 co-regulate senescence of trophoblast cells

**Fig. 4.**
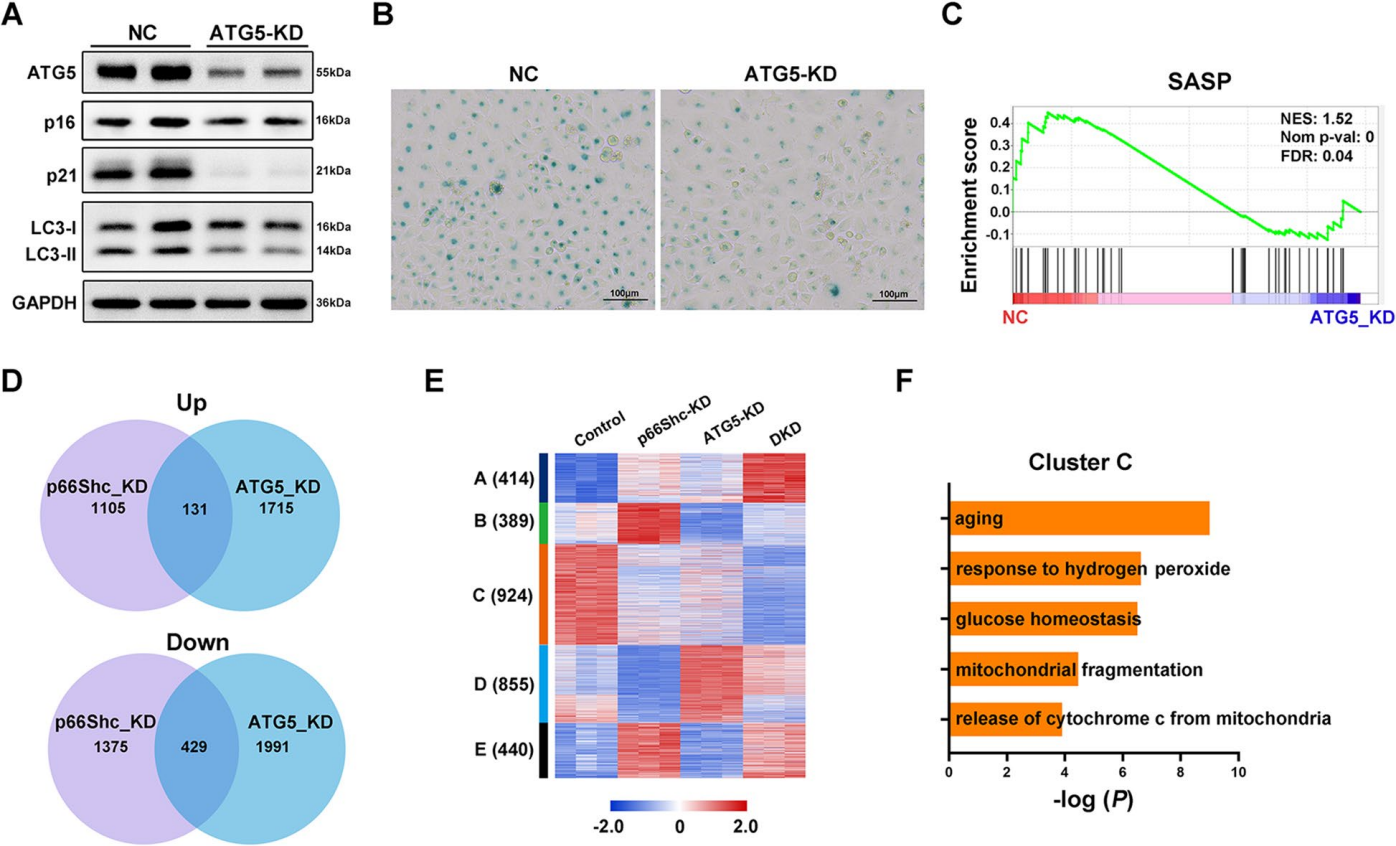
p66Shc and ATG5 co-regulate senescence of trophoblast cells. **A** Western blot images of protein levels of ATG5, p16, p21 and LC3 in control and ATG5-KD cells. **B** SA-β-gal staining showing cell senescence of control and ATG5-KD cells. **C** Gene set enrichment analysis of SASP gene signature in control and ATG5-KD cells. **D** Venn diagram illustrates the number of differentially expressed genes overlapped between p66Shc-KD and ATG5-KD cells. **E** K-means clustering heatmap shows expressions patterns of differentially expressed genes upon p66Shc-KD. **F** Gene ontology enrichment analysis of genes in cluster C

We next explored whether p66Shc regulates cellular senescence through autophagy. We used small interfering RNA to ablate ATG5 expression (ATG5-KD) to inhibit autophagy, and dramatic reductions of protein level of ATG5 and ratio of LC3II/LC3I were observed (Fig. 4A). Deletion of ATG5 leads to decreased protein levels of p16 and p21, and the SA β-gal activity (Fig. 4A-B).

We then performed RNA-seq in ATG5-KD and ATG5-p66Shc double-KD (DKD) cells. GSEA also revealed that the inhibition of ATG5 repressed genes associated with SASP (Fig. 4C), indicating autophagy supports the production of a robust SASP. Interestingly, we found a set of genes was co-dysregulated upon either p66Shc or ATG5 knockdown (Fig. 4D), demonstrating they shared common downstream targets. To better understand the gene expression changes affected by p66Shc and ATG5, we performed k-means clustering of genes differentially expressed by p66Shc knockdown across p66Shc-KD, ATG5-KD and ATG5-p66Shc DKD cells. Notably, these genes showed five distinct expression patterns (Fig. 4E), in which genes belong to cluster C were focused as they were downregulated upon either p66Shc-KD or ATG5-KD, and displayed more repressed patterns in DKD cells. These genes were significantly enriched for biological processes including aging, oxidative response and mitochondrial homeostasis (Fig. 4F). Together, these findings demonstrated cellular senescence is co-regulated by p66Shc and ATG5, and p66Shc regulates senescence through autophagy.

### p66Shc regulates MAMs formation and PINK1-PRKN-mediated mitophagy in high glucose

**Fig. 5.**
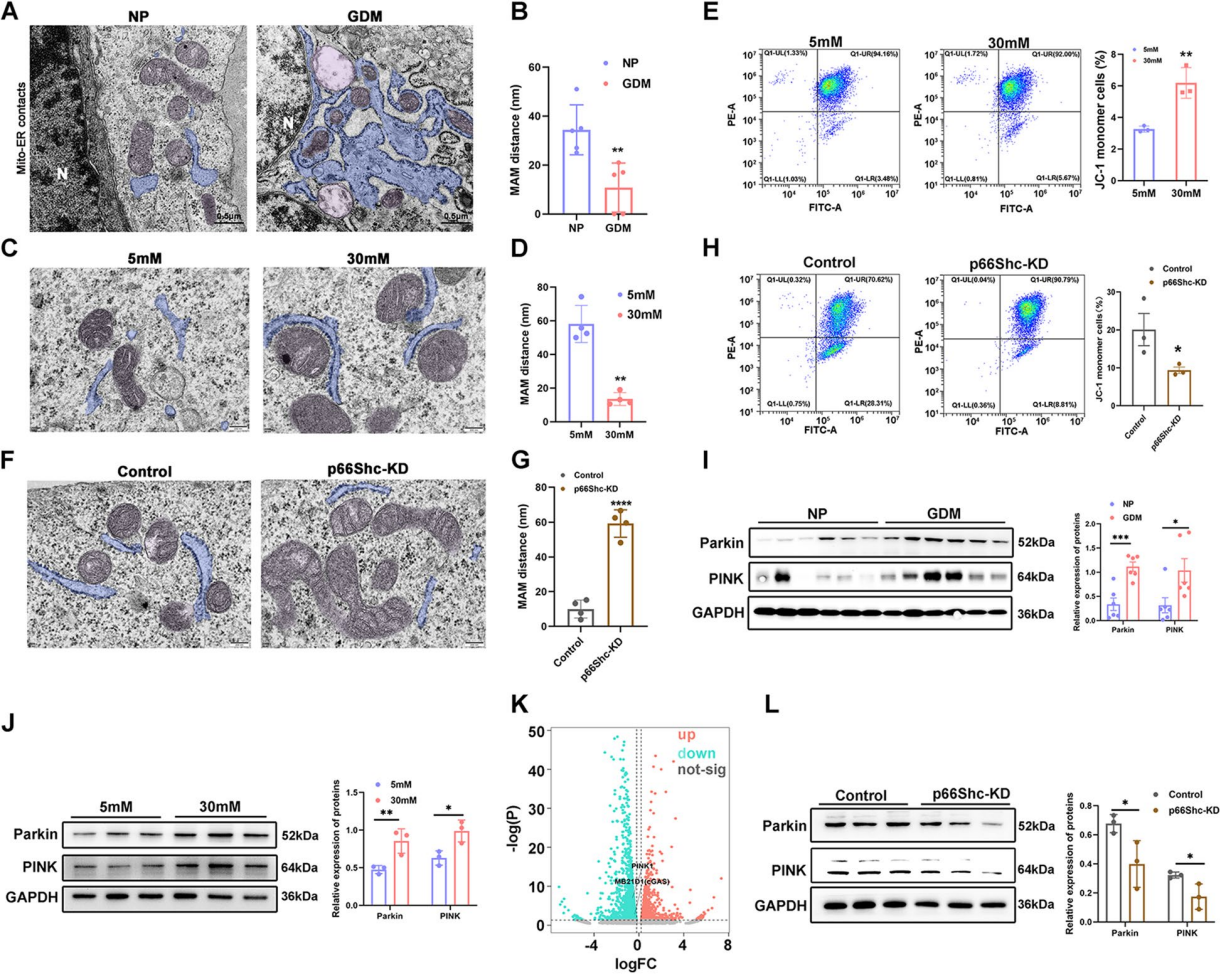
p66Shc regulates formation of MAM and mitophagy. **A**-**B** Representative TEM images **A** and quantifications **B** of mitochondrial ultrastructural changes in NP and GDM placentae, mitochondria (in purple) in close contact with ER (in blue) in which distance ≤ 25 nm were considered as MAM sites. Scale bar, 0.5 μm; N, nucleus. **P < 0.01. **C**-**D** Representative TEM images **C** and quantifications **D** of mitochondria (in purple) in close contact with ER (in blue) in which distances ≤ 25 nm was considered as contacts in 5 mM and 30 mM glucose treated HTR8/SVneo cells. Scale bar, 200 nm. **P < 0.01. **E** JC-1-based flow cytometry assay images and quantifications of fluorescence intensity distribution of 5 mM or 30 mM glucose treated HTR8/SVneo cells. **P < 0.01. **F**-**G** Representative TEM images **F** and quantifications **G** of Mito-ER contacts distance in control and p66Shc-KD cells. Scale bar, 200 nm. ****P < 0.0001. **H** JC-1-based flow cytometry assay images and quantifications of fluorescence intensity distribution of control or p66Shc-KD cells. *P < 0.05. **I**-**J** Western blot images and quantifications of protein levels of Parkin and PINK in (I) NP and GDM placentae and **J** 5 mM and 30 mM glucose treated HTR8/SVneo cells. *P < 0.05; **P < 0.01; ****P < 0.0001. **K** Volcano plot showing the mRNA expression changes upon p66Shc knockdown. **L** Western blot images and quantifications of protein levels of Parkin and PINK in control and p66Shc-KD cells. *P < 0.05

As MAMs are crucial for autophagy initiation and mitochondrial function, we next explored whether p66shc is associated with MAMs formation. We first carried out TEM analysis to examine mitochondrial ultrastructural changes in the syncytiotrophoblast cells of GDM patients. We showed that Mito-surface was covered with ER, and Mito dissolved into vacuole in syncytiotrophoblast cells of GDM placentae (Fig. 5A-B), as compared to our previous observations in the NP group [3], indicating a significantly increased MAM number and damaged Mito in syncytiotrophoblast cells of high glucose. Likewise, a closer intermembrane distance of Mito-ER and significantly a larger MAM coupling surface were observed in HTR8/SVneo and Bewo cells exposed to high glucose (Fig. 5C-D and Figure S4), accompanied with a significant reduction in the MMP (Fig. 5E), indicating mitochondrial depolarization of trophoblast cells exposed to high glucose. In contrast, p66Shc-KD cells displayed markedly enlarged the Mito-ER distance and significantly reduced the Mito-surface covered to the ER (Fig. 5F-G), and enhanced MMP (Fig. 5H) in high glucose. These results suggested that p66Shc regulates MAMs site formation in high glucose stress.

Previous studies have shown that the formation of MAMs sites is enhanced by PINK following mitochondrial depolarization, and PINK1-PRKN-mediated mitophagy occurs upon loss of MMP [17, 18]. We assessed the expression of PINK1 and Parkin in high glucose treated HTR8/SVneo cells or p66Shc-KD cells. Western blot results demonstrated an upregulation of PINK1 and Parkin in placentae from GDM patients, as well as in high glucose treated HTR8/SVneo and Bewo cells (Fig. 5I-J and Figure S5A). Additionally, our RNA-seq revealed *PINK* mRNA was significantly reduced upon p66Shc knockdown (Fig. 5K), indicating p66Shc regulates mitophagy in trophoblast cells. Likewise, a decrease of PINK and Parkin were pronounced after 24 hours of high glucose treatment in the whole lysate of p66Shc-KD cells compared with control (Fig. 5L). Interestingly, knockdown of p66Shc did not result in a reduction in the expression of PINK and Parkin under normal glucose condition (Figure S5B). These results implied that p66Shc knockdown exclusively enhances mitochondrial function during cellular stress.

### p66Shc modulates cGAS/STING-mediated autophagosome formation in trophoblast cells

**Fig. 6:**
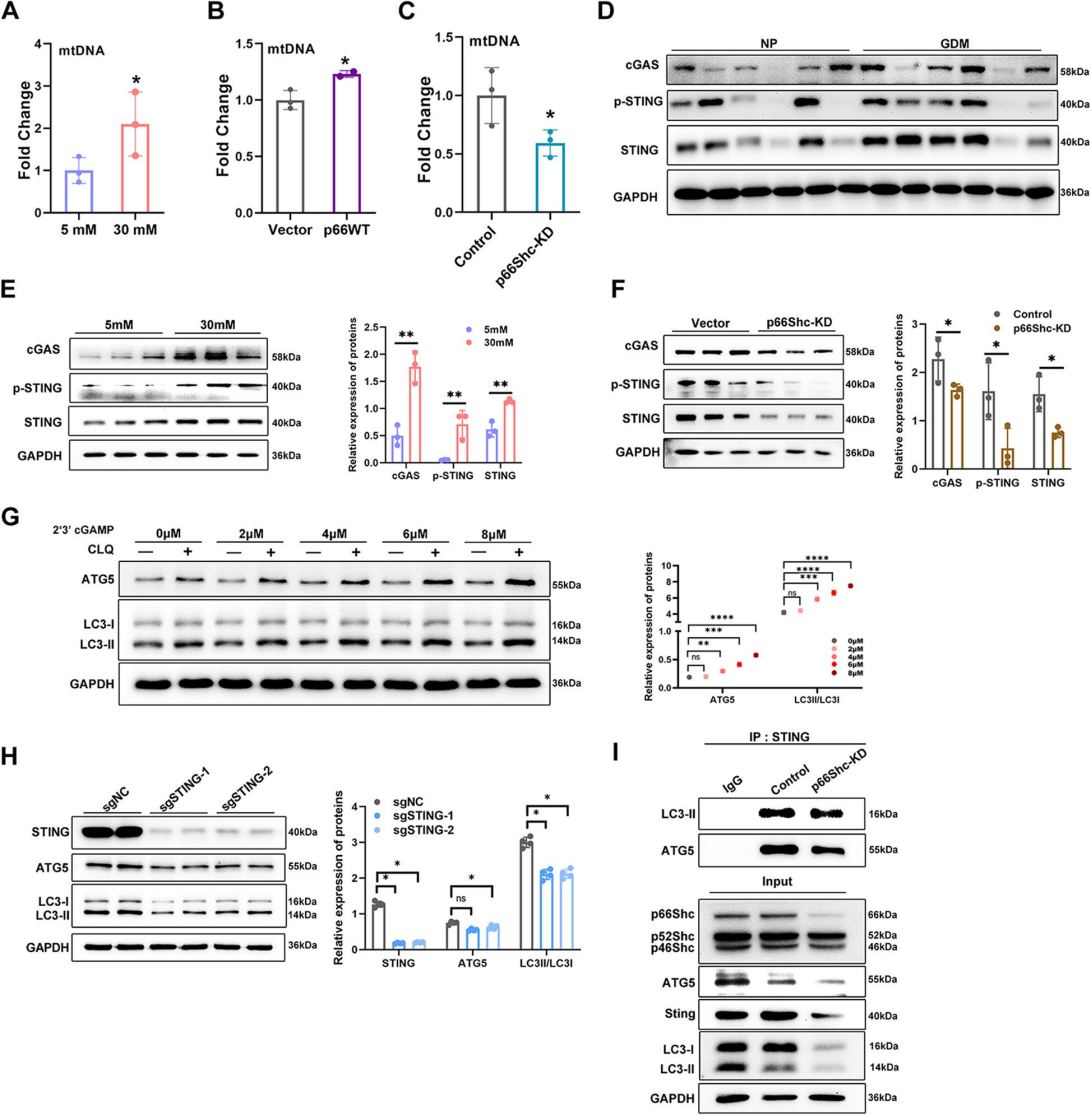
p66Shc mediated autophagy through cGAS/STING pathway **A**-**C** qRT-PCR analysis of relative cytosolic mtDNA levels in **A** 5 mM or 30 mM glucose treated HTR8/SVneo cells, **B** Control and p66WT overexpressed HTR8/SVneo cells, **C** Control and p66Shc-KD cells. *P < 0.05. **D** Western blot images of protein levels of cGAS, p-STING and STING in normal and GDM placentae. **E** Western blot images and quantifications of protein levels of cGAS, p-STING and STING in 5 mM or 30 mM glucose treated HTR8/SVneo cells. **P < 0.01. **F** Western blot images and quantifications of protein levels of cGAS, p-STING and STING in control and p66Shc-KD cells. *P < 0.05. **G** Western blot images and quantifications of protein levels of p-STING, STING and LC3II in HTR8/SVneo cells treated with 2’3’-cGAMP and Chloroquine. **P < 0.01, ***P < 0.001, ****P < 0.0001. **H** Western blot images and quantifications of protein levels of STING, LC3 and ATG5 in control and STING-KO HTR8/SVneo cells. *P < 0.05. **I** Co-immunoprecipitation coupled western blot images demonstrate the interactions between STING and LC3 or ATG5 in both control and p66Shc-KD cells

It has been reported that mitochondrial permeability transition pore (mPTP) is involved in mediating mtDNA release under different cell stress [13]. Our present study observed loss of MMP (Fig. 5E) and upregulation of ROS (Fig. 1F), which indicated opening of the mPTP [19]. As speculated, high glucose significantly augmented the release of mtDNA into the cytoplasm (Fig. 6A). Furthermore, p66Shc overexpression-mediated mtDNA accumulation in cytoplasm is significantly inhibited following p66Shc knock down (Fig. 6B-C).

Cytosolic mtDNA is a known activator of cGAS/STING signaling pathway, and an inductor of autophagy [13]. Consistent with a role of cytosolic mtDNA in the activation of cGAS/STING pathway, we found increased cGAS, STING and p-STING in GDM placentae and high glucose treated HTR8/SVneo cells (Fig. 6D-E). We also observed activated STING in Bewo cells (Figure S5C). Our RNA-seq revealed cGAS (MB21D1) mRNA was significantly reduced upon p66Shc knockdown (Fig. 5K). In addition, decreased cGAS, STING and p-STING were observed in p66Shc-KD cells (Fig. 6F). These results suggested that p66Shc induced mtDNA release and cGAS/STING signaling activation in trophoblast cells of high glucose.

Recently, the cGAS/STING was found to promote autophagy [11, 13]. Hence, we investigated whether cGAS/STING pathway is involved in p66Shc mediated autophagy in trophoblast of high glucose. HTR8/SVneo cells were subjected to a 24-hour treatment with 2’3’-cGAMP, a cyclic dinucleotide secondary messenger that binds to and activates STING. In response to 2’3’-cGAMP stimulation, we found a concentration-dependent increase in LC3 conversion and ATG5 expression (Fig. 6G). Furthermore, we knocked out STING with CRISPR/Cas9 to explore the role of STING in autophagy of trophoblast cells. Notably, STING knockout impedes LC3 conversion and ATG5 expression in vitro (Fig. 6H). It has been suggested that STING-LC3II colocalization is required for enhanced autophagy [11]. We further investigated the potential contribution of p66Shc in mediating cGAS/STING-dependent autophagy. The association between STING and LC3II were observed, however, lower levels of interaction of endogenous LC3II and ATG5 were detected following co-immunoprecipitation of STING from p66Shc-KD cells (Fig. 6I). Collectively, these findings suggest that knockdown of p66Shc disrupts the activation of STING and impairs the interaction between STING and LC3II, as well as between STING and ATG5, thereby contributing to a reduction in autophagosome formation in trophoblast cells.

## Discussion

Accumulating evidence indicates that pregnancy accompanied by GDM is associated with increased oxidative stress levels compared with normal pregnancy [20, 21]. GMD also causes mitochondrial dysfunction in the placental endothelium and trophoblast [22]. In the mitochondrial intermembrane space, p66Shc act as an oxidoreductase that produces ROS [23], overproduced ROS disrupt the balance between oxidation and antioxidation, ultimately lead to tissue damage in GDM [6]. In this study, we demonstrate up-regulation of p66Shc mediated autophagy and mitophagy, as well as mitochondrial dysfunction in GDM placentae, several researches support our conclusions [5, 6, 22, 24]. Moreover, we present novel evidence that p66Shc is implicated in the formation of MAMs in trophoblast cells, potentially mediating autophagosome initiation. Additionally, p66Shc is involved in the release of mtDNA into the cytoplasm, leading to cGAS/STING activation and subsequent induction of a cellular senescence phenotype. Lastly, p66Shc disrupts the association between STING and LC3II, as well as between STING and ATG5, resulting in reduced levels of autophagic flux.

Mitochondrion is the primary cellular organelle responsible for generating ROS, and excessive ROS is detrimental and associated with GDM. The MnSOD is an antioxidative enzyme present in the mitochondria, utilized as a biomarker for oxidative stress [25, 26]. Accompanied by a high level of serum Mn-SOD in GDM patients, we hypothesize that an imbalance between oxidants and antioxidants leads to cellular senescence in trophoblast cells. As expected, trophoblast demonstrated enhanced p21 and several SASP markers, but also showed unchanged p16 and few other SASP markers. It maybe accords to the evidence that p21 is mainly activated early during the evolution of senescence, whereas p16 maintains cellular senescence [27]. However, knockdown of p66Shc rescues high glucose-induced cellular senescence, potentially due to a reduction in autophagic activity.

The role of autophagy in placentae of GDM remains controversial [28], our previous studies showed increased autophagy in trophoblast cell both in vivo and in vitro [3]. In present study, we found Mito-surface was enveloped by ER, indicating an augmented abundance of MAMs and the initiation of autophagy. The mitochondrial pool of p66SHC not only leads to mitochondrial dysfunctions but also results in dissipation of the MMP, this may not be sufficient to sustain its execution for p66Shc-mediated autophagy in trophoblast cells. Indeed, the functionality of this process relies on the essential requirement for direct binding between mitochondria-associated p66Shc and LC3-II [5], regardless of whether it occurs in B cells or trophoblast cells. Surprisingly, in addition to facilitating the engulfment of damaged mitochondria by pre-formed phagophore membranes autophagosomes, this interaction is required for the formation of MAMs. Although MAMs are involved in energy stress, tumorigenesis, neurodegenerative et al. [10, 29, 30], this is the first study to demonstrate that p66Shc regulates the formation of MAMs in trophoblast cell of high glucose. One possible mechanism for this is the oxidative stress increased mitochondrial translocation of p66Shc, therefore result in damaged mitochondria and enhanced MAM proteins expression, such as PINK and Parkin. Certainly, further investigations should be conducted to elucidate the underlying molecular mechanisms through which p66Shc facilitates the formation of MAM in the placentae of patients with GDM.

Besides, cytoplasmic mtDNA is another trigger of autophagy in trophoblast cells of GDM. Cytoplasmic DNA accumulation can trigger aberrant activation of cGAS/STING and STING-mediated autophagy [11, 13]. Our present study demonstrated that decreased MMP by p66Shc overexpression and its mitochondrial translocation may disrupt mPTP and induce cytosolic mtDNA stress in trophoblast cell. This leads to a substantial upregulation of STING phosphorylation and the induction of autophagic flux. Conversely, knockdown of p66Shc significantly attenuated cytosolic mtDNA and STING activation in trophoblast cells exposed to high glucose. We further observed that p66Shc disrupted the interaction between STING and LC3-II, as well as between STING and ATG5, indicating p66Shc plays a direct role in STING-mediated autophagosomes formation in trophoblast cells. Although co-localization of STING and LC3II has been previously reported, the interaction between STING and ATG5 was discovered for the first time. ATG5 was used to observe autophagosome formation, it binds only to the isolation membrane and detaches from the membrane after autophagosome formation completion, which was a specific marker of the initial stages of autophagosome formation. Therefore, STING is likely to be involved in the entire process of autophagy, spanning from the initial formation of the isolation membrane until fusion occurs between the autophagosome and lysosome. These findings above suggest that p66Shc is not only indirectly implicated in the initiation of autophagy but may help by stabilizing active autophagic flux in trophoblast.

## Conclusion

**Fig. 7:**
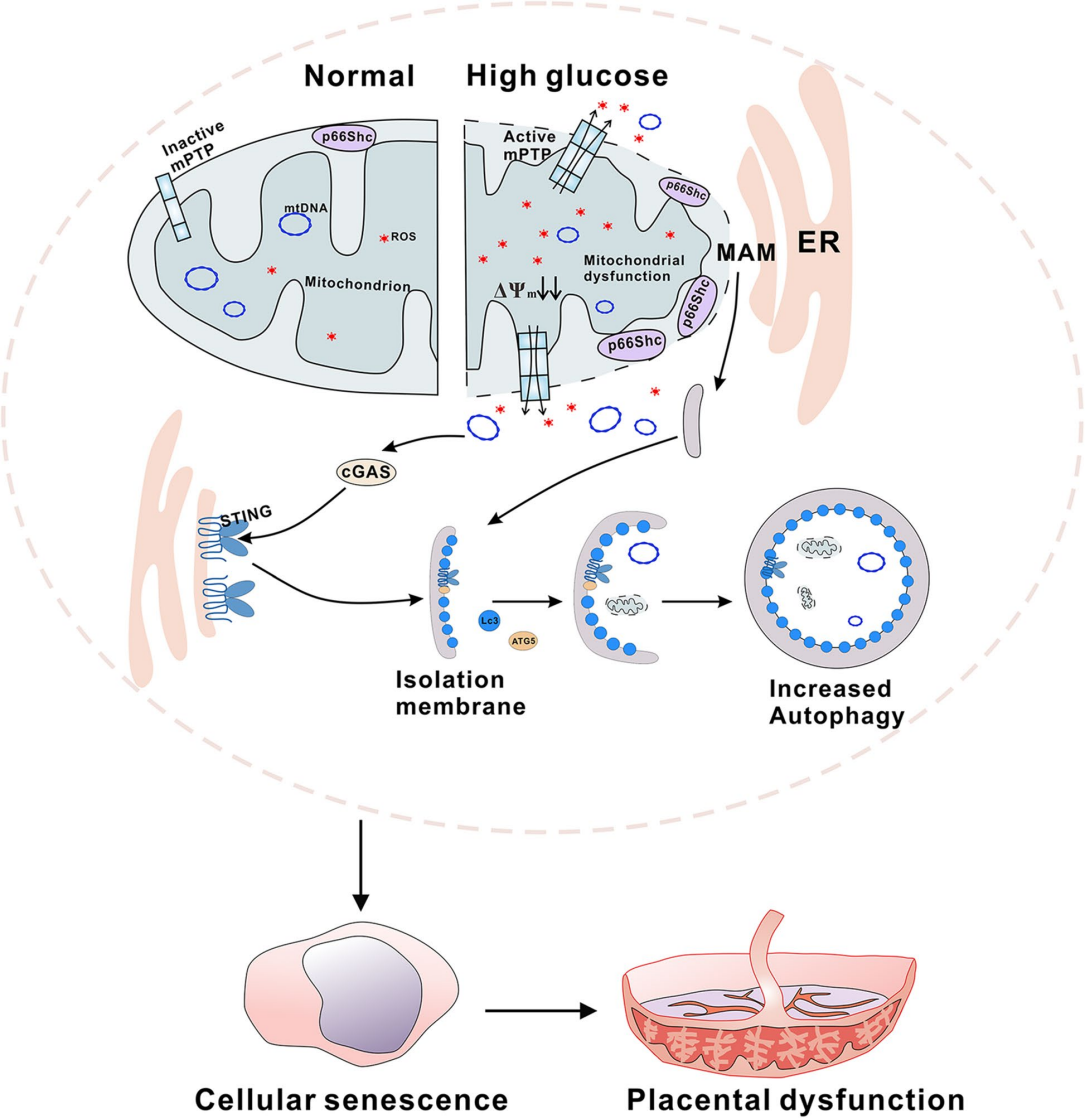
A model of p66Shc orchestrates autophagy via cGAS-STING signaling in trophoblast cells of GDM.Step 1, High glucose induces an upregulation of p66Shc expression and its translocation to the mitochondria, resulting in the accumulation of ROS and a reduction in MMP. This leads to activation of the mPTP, which subsequently causes the release of ROS and mtDNA into the cytoplasm. Step 2, p66Shc translocases into mitochondrial intermembrane space promotes the formation of MAMs, which serves as a membrane source for LC3 recruitment and lipidation through an ATG5 dependent mechanism. Step 3, The cytoplasmic mtDNA triggers and activation of STING, leading to the subsequent translocation of STING from the endoplasmic reticulum to the isolation membrane. LC3-positive membranes target DNA and dysfunctional mitochondrion to autophagosomes, which results in increased autophagy in trophoblast cells. Besides, this dysregulation ultimately impairs trophoblast cell function and potentially compromises placental integrity in patients with GDM

In present study, we observed that overexpression and mitochondrial translocation of p66Shc led to mtDNA stress, resulting in mitochondrial dysfunction and formation of MAMs that initiate autophagy. Subsequently, cGAS/STING pathway involved autophagy was also activated in trophoblast cells (Fig. 7). Our findings suggest an important contribution of the p66Shc orchestrates autophagy through MAMs and the cGAS/STING signaling pathway, highlighting the potential therapeutic strategy of targeted suppression of p66Shc-mediated autophagy for managing placental dysfunction in patients with GDM.

### Supplementary Information

**Additional file1: Figure S1**. The mRNA expression of *p66Shc* and its translocation to mitochondrial in Bewo cells. (A) Western blot images of protein expressions of p66Shc in cytosol and mitochondria in 5 mM or 30 mM glucose treated Bewo cells. (B) qRT-PCR analysis of *p66Shc* mRNA expression in NP and GDM placentae. *P < 0.05. (C) qRT-PCR analysis of *p66Shc* mRNA expression in 5 mM or 30 mM glucose treated HTR8/SVneo cells. ***P < 0.001. (D) Fluorescence microscope images of co-localization of p66Shc (green) with mitochondria (red) in 5 mM or 30 mM glucose treated Bewo cells. Scale bar, 20 μm.

**Additional file 2: Figure S2**. Maternal serum Mn-SOD associate with BMI rather than age. (A) Box plot shows the levels of BMI between NP (*n* = 281) and GDM patients (*n* = 1037). (B) Correlation analysis between BMI and serum Mn-SOD. (C) Correlation analysis of serum Mn-SOD levels with age.

**Additional file 3:** **Figure S3**. Schematic structural representations of mutant variants of p66Shc and levels of SASP proteins in placentae and Bewo cells. (A) Schematic structural representations of two isoforms for p66Shc mutations (SC and QQ) and the schematic amino acid sequence representations of p66Shc mutations. (B) Western blot images and quantifications of SASP protein levels of p16, MCP1, MMP3 and PAI in NP and GDM placentae. (C) Western blot images and quantifications of SASP protein levels of p16 and p21 in Bewo cells. **P < 0.01, ***P < 0.001.

**Additional file 4: Figure S4**. The formation of MAM in high glucose treated Bewo cells. Representative TEM images of mitochondria (in purple) in close contact with ER (in blue) in 5 mM and 30 mM glucose treated Bewo cells. Scale bar, 200 nm.

**Additional file 5: Figure S5**. The protein expression in Bewo or p66Shc-KD cells (A) Western blot images and quantifications of protein levels of Parkin and PINK in high glucose treated Bewo cells and (B) in normal glucose (NG) treated control or p66Shc-KD cells. (C) Western blot images and quantifications of protein levels of cGAS, p-STING and STING in high glucose treated Bewo cells. *P < 0.05, **P < 0.01.

**Additional file 6.**

**Additional file 7.**

**Additional file 8.**

## Data Availability

The datasets used and/or analyzed during the current study are available from the corresponding author on reasonable request.
