# Supplementary figures and images for: High glucose-induced p66Shc mitochondrial translocation regulates autophagy initiation and autophagosome formation in syncytiotrophoblast and extravillous trophoblast
Source: Cell Commun Signal. 2024 Apr 20;22:234. doi: 10.1186/s12964-024-01621-x (PMC11031965; doi:10.1186/s12964-024-01621-x)

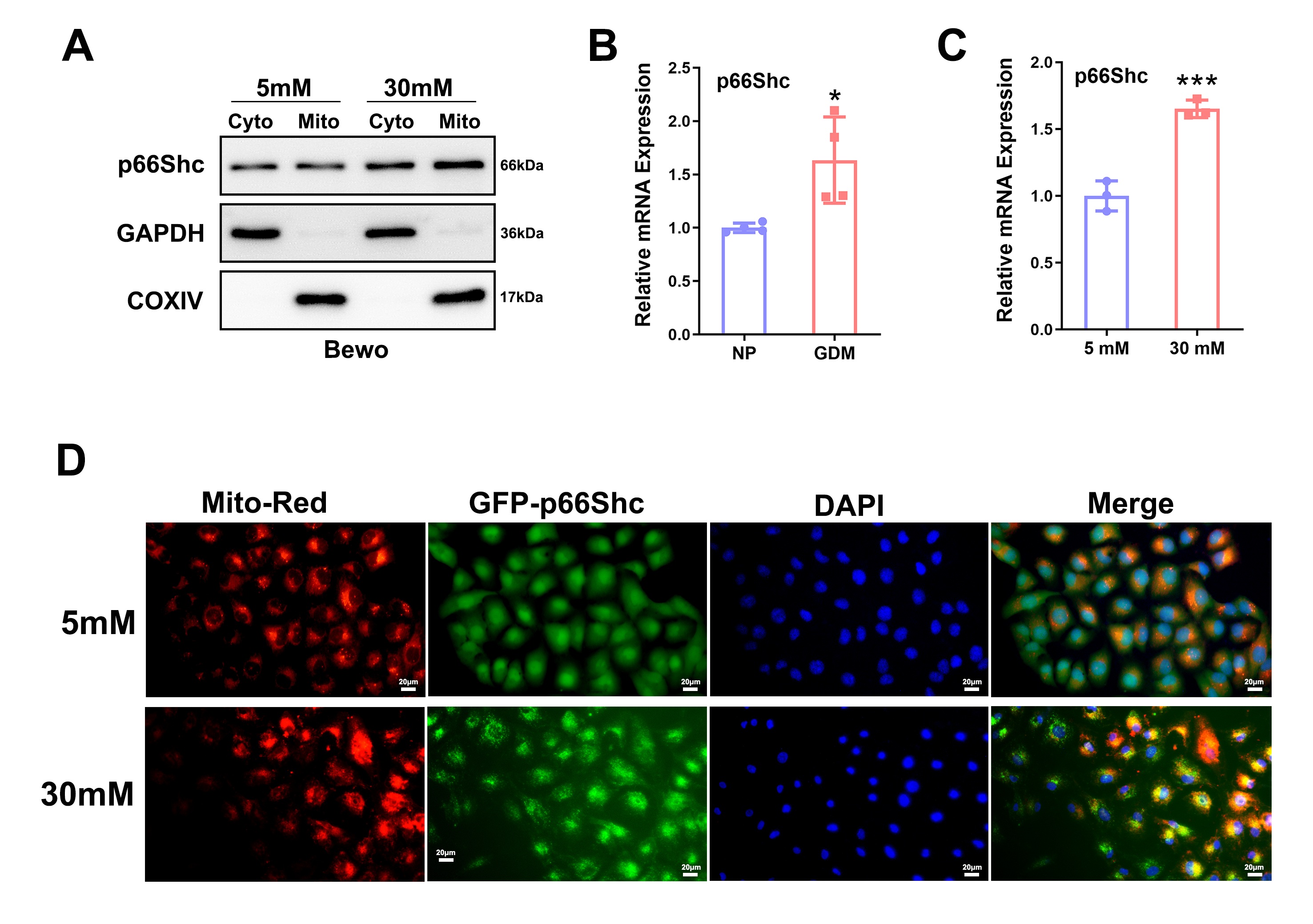

Supplement: Supplementary file 1 — Additional file1: Figure S1. The mRNA expression of p66Shc and its translocation to mitochondrial in Bewo cells. (A) Western blot images of protein expressions of p66Shc in cytosol and mitochondria in 5 mM or 30 mM glucose treated Bewo cells. (B) qRT-PCR analysis of p66Shc mRNA expression in NP and GDM placentae. *P < 0.05. (C) qRT-PCR analysis of p66Shc mRNA expression in 5 mM or 30 mM glucose treated HTR8/SVneo cells. ***P < 0.001. (D) Fluorescence microscope images of co-localization of p66Shc (green) with mitochondria (red) in 5 mM or 30 mM glucose treated Bewo cells. Scale bar, 20 μm. [file 12964_2024_1621_MOESM1_ESM.tif]

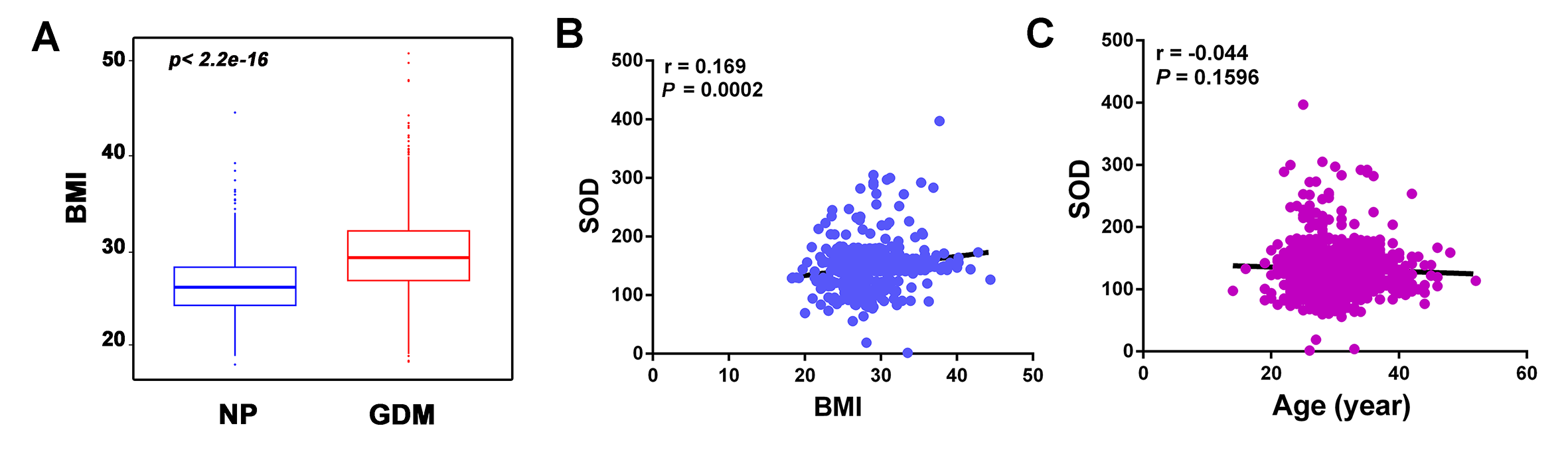

Supplement: Supplementary file 2 — Additional file 2: Figure S2. Maternal serum Mn-SOD associate with BMI rather than age. (A) Box plot shows the levels of BMI between NP (n = 281) and GDM patients (n = 1037). (B) Correlation analysis between BMI and serum Mn-SOD. (C) Correlation analysis of serum Mn-SOD levels with age. [file 12964_2024_1621_MOESM2_ESM.tif]

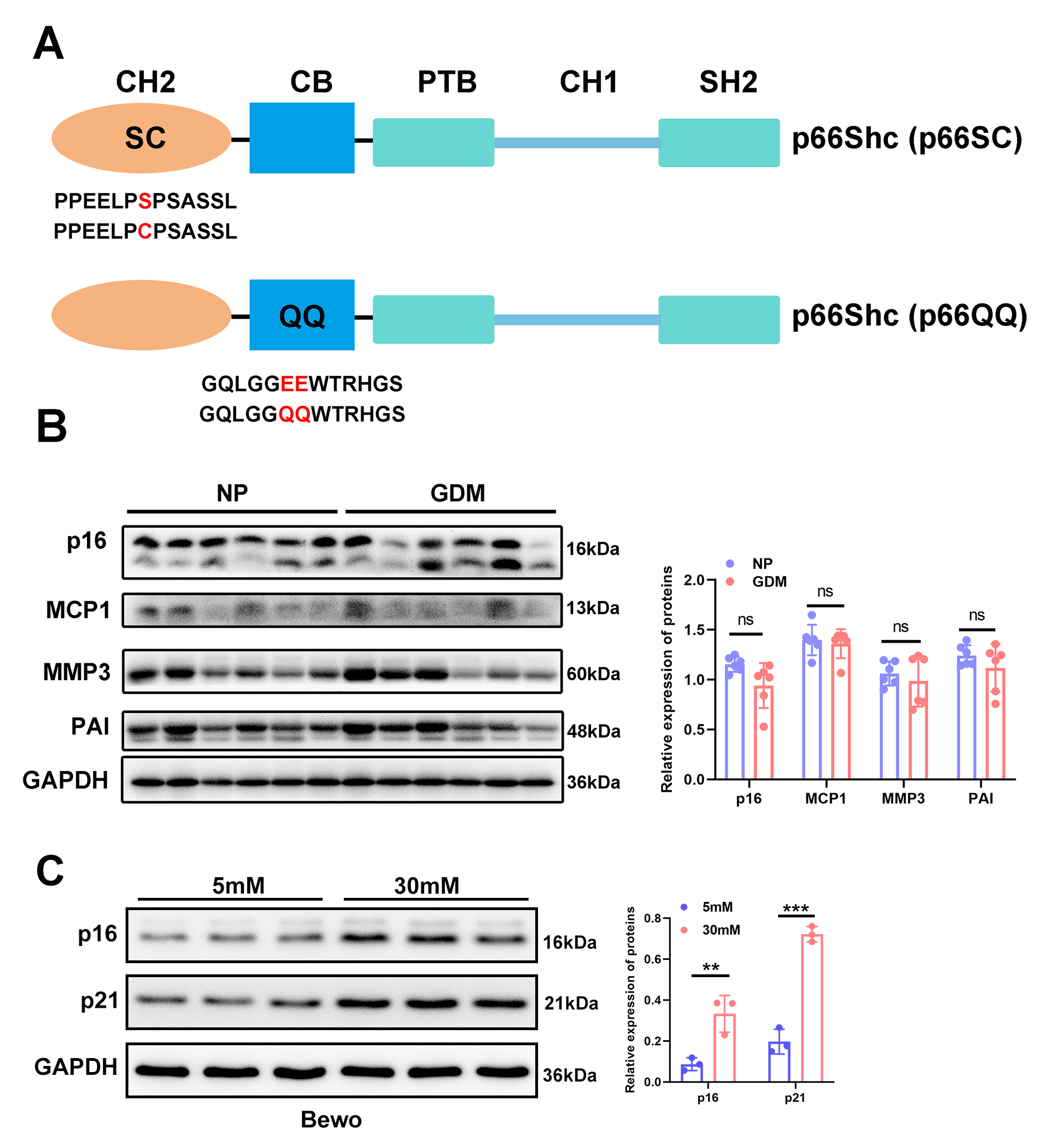

Supplement: Supplementary file 3 — Additional file 3: Figure S3. Schematic structural representations of mutant variants of p66Shc and levels of SASP proteins in placentae and Bewo cells. (A) Schematic structural representations of two isoforms for p66Shc mutations (SC and QQ) and the schematic amino acid sequence representations of p66Shc mutations. (B) Western blot images and quantifications of SASP protein levels of p16, MCP1, MMP3 and PAI in NP and GDM placentae. (C) Western blot images and quantifications of SASP protein levels of p16 and p21 in Bewo cells. **P < 0.01, ***P < 0.001. [file 12964_2024_1621_MOESM3_ESM.tif]

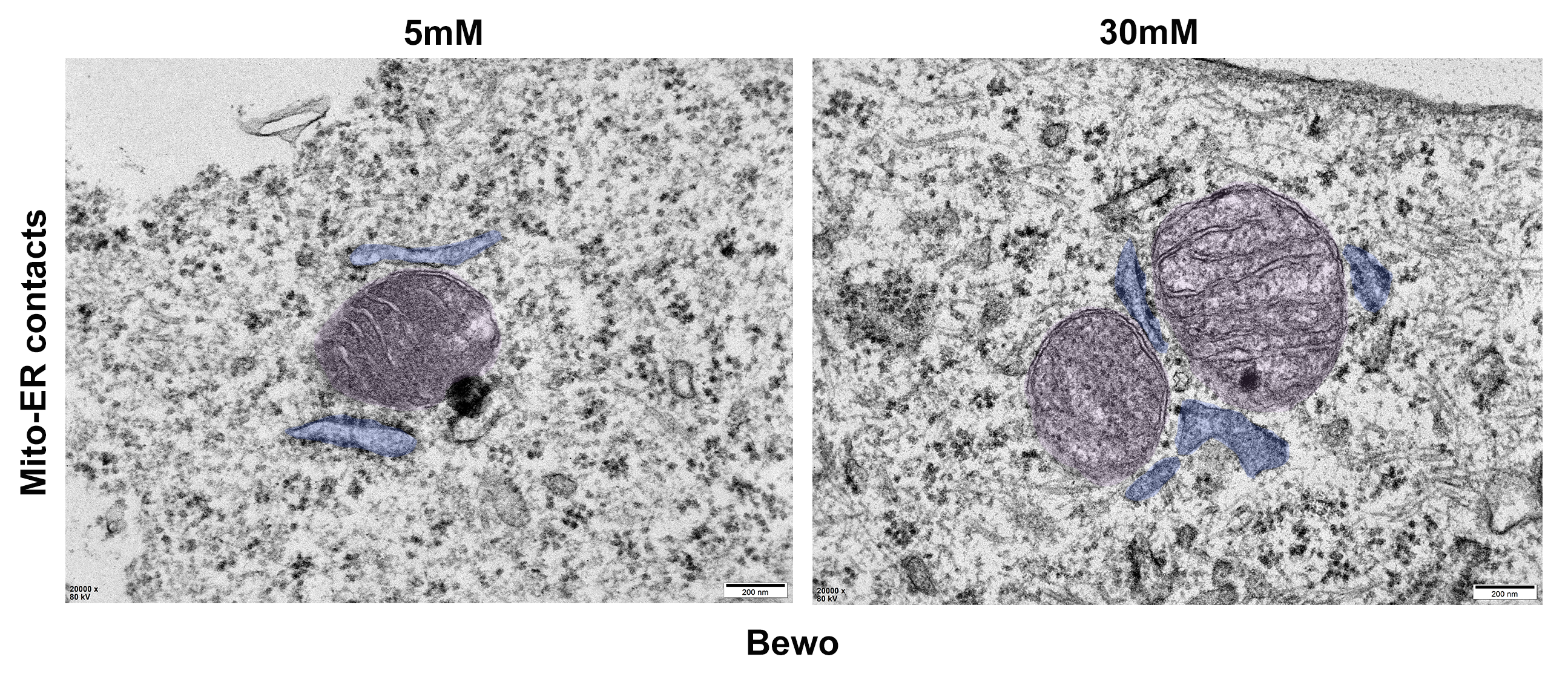

Supplement: Supplementary file 4 — Additional file 4: Figure S4. The formation of MAM in high glucose treated Bewo cells. Representative TEM images of mitochondria (in purple) in close contact with ER (in blue) in 5 mM and 30 mM glucose treated Bewo cells. Scale bar, 200 nm. [file 12964_2024_1621_MOESM4_ESM.tif]

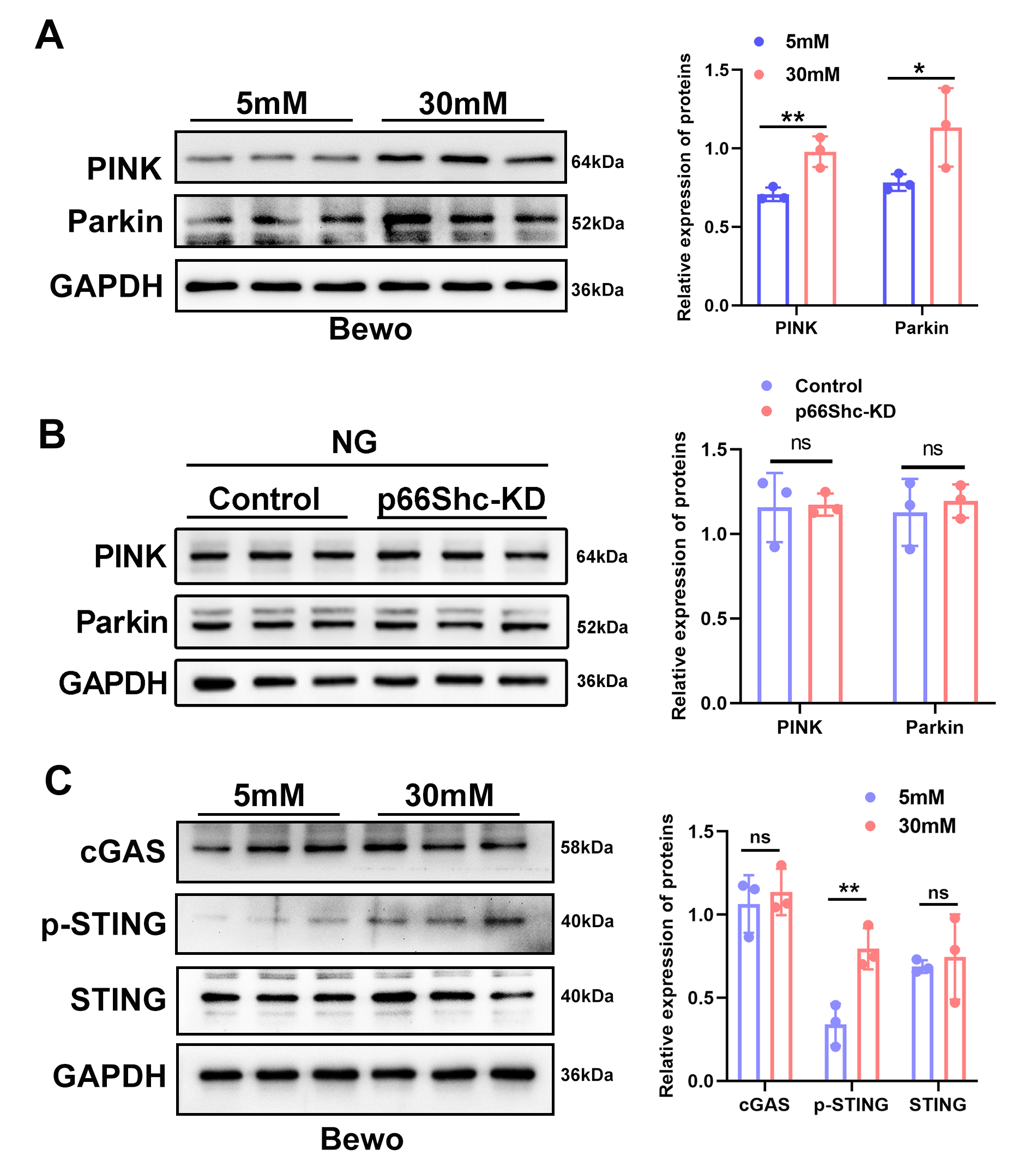

Supplement: Supplementary file 5 — Additional file 5: Figure S5. The protein expression in Bewo or p66Shc-KD cells (A) Western blot images and quantifications of protein levels of Parkin and PINK in high glucose treated Bewo cells and (B) in normal glucose (NG) treated control or p66Shc-KD cells. (C) Western blot images and quantifications of protein levels of cGAS, p-STING and STING in high glucose treated Bewo cells. *P < 0.05, **P < 0.01. [file 12964_2024_1621_MOESM5_ESM.tif]
